# Human T Cell Differentiation Negatively Regulates Telomerase Expression Resulting in Reduced Activation-Induced Proliferation and Survival

**DOI:** 10.3389/fimmu.2019.01993

**Published:** 2019-08-21

**Authors:** Michael S. Patrick, Nai-Lin Cheng, Jaekwan Kim, Jie An, Fangyuan Dong, Qian Yang, Iris Zou, Nan-ping Weng

**Affiliations:** Laboratory of Molecular Biology and Immunology, National Institute on Aging, National Institutes of Health, Baltimore, MD, United States

**Keywords:** T lymphocytes, T cell subsets, telomerase, hTERT, alternative splicing, differentiation, proliferation, aging

## Abstract

Maintenance of telomeres is essential for preserving T cell proliferative responses yet the precise role of telomerase in human T cell differentiation, function, and aging is not fully understood. Here we analyzed human telomerase reverse transcriptase (hTERT) expression and telomerase activity in six T cell subsets from 111 human adults and found that levels of hTERT mRNA and telomerase activity had an ordered decrease from naïve (T_N_) to central memory (T_CM_) to effector memory (T_EM_) cells and were higher in CD4^+^ than their corresponding CD8^+^ subsets. This differentiation-related reduction of hTERT mRNA and telomerase activity was preserved after activation. Furthermore, the levels of hTERT mRNA and telomerase activity were positively correlated with the degree of activation-induced proliferation and survival of T cells *in vitro*. Partial knockdown of hTERT by an anti-sense oligo in naïve CD4^+^ cells led to a modest but significant reduction of cell proliferation. Finally, we found that activation-induced levels of telomerase activity in CD4^+^ T_N_ and T_CM_ cells were significantly lower in old than in young subjects. These findings reveal that hTERT/telomerase expression progressively declines during T cell differentiation and age-associated reduction of activation-induced expression of hTERT/telomerase mainly affects naïve CD4^+^ T cells and suggest that enhancing telomerase activity could be a strategy to improve T cell function in the elderly.

## Introduction

T cell differentiation is an essential process for establishing long lasting immunity. During this process, naïve T cells differentiate into effector cells to combat pathogens and become memory T cells for long term protection (1). As a consequence of differentiation, T cells acquire distinct, and specialized functions in each specific subset of differentiated T cells (2, 3). Human CD4^+^ and CD8^+^ T cells in peripheral blood are composed of mainly naive (T_N_), central (T_CM_), and effector (T_EM_) memory T cells as defined by cell surface expression of CD62L/CCR7 and CD45RA (4). T_CM_ and T_EM_ are two major types of memory T cells and some evidence suggests that T_CM_ can further differentiate to become T_EM_ in response to antigen and cytokines (5, 6). Recent transcriptome analyses reveal distinct gene expression changes associated with human T cell differentiation and their functional specialization (7–9). In contrast to well-documented gain of function during T cell differentiation, loss of function during T cell differentiation has not been fully characterized.

Human telomerase enzyme consists of two core components: human telomerase reverse transcriptase (hTERT) and telomerase RNA template (hTERC) (10). hTERT is a rate-limiting component of telomerase and is regulated at several levels of transcription, post-transcription (alternative splicing), and post-translational modifications (11, 12). Unlike most mature somatic cells that do not express telomerase, lymphocytes have the ability to regulate telomerase expression during lineage development and activation (13). Freshly isolated resting human T cells express hTERT mRNA but little to no telomerase activity (14–16). Explanations for this hTERT mRNA and telomerase activity discordance include a requirement for post-translational modifications of hTERT for telomerase activity (17) or the exclusive presence of alternatively spliced products (ASP) of hTERT in resting T cells (18) which results in loss of hTERT function (19). After activation, T cells rapidly upregulate hTERT mRNA and telomerase activity to ensure robust cell division and differentiation (20). Furthermore, increased telomerase expression extends the replicative lifespan of T cells (21). However, the extent of hTERT ASP expression in human resting and activated T cells and their role in T cell differentiation and aging have not been fully characterized.

Loss of telomere length during T cell differentiation has long been recognized, as human T_N_ cells have longer average telomere lengths, greater *in vitro* proliferative capacity, and higher hTERT mRNA level than memory T cells (22–24). Within the memory compartment, T_CM_ cells have greater proliferative capacity, and telomere length than those of T_EM_ cells (4). These observations suggest that T cell differentiation leads to telomere shortening and reduced cell proliferative potential. But it has not been determined whether hTERT/telomerase is also altered during CD4^+^ and CD8^+^ T cell differentiation from T_N_ to T_CM_ to T_EM_ and whether regulation of hTERT/telomerase differs in CD4^+^ and CD8^+^ T cells.

Aging has a detrimental impact on T cell generation and function (25, 26). *In vitro* long term culture of primary human T cells shows reduced telomere length and reduced levels of hTERT mRNA and telomerase activity (20, 24). *In vivo* telomere length attrition in T cells with age has also been reported (27, 28). T cells with shorter telomeres are not only a biomarker of T cell aging, but also associated with reduced T cell proliferation (29). Furthermore, reduced telomerase activity in T and B cells with age has been reported (30). In addition, hTERT expression has been positively associated with an increase in influenza-specific memory B cells in response to influenza vaccination in the elderly (31). Collectively, these findings suggest that age affects telomere maintenance and telomerase expression in lymphocytes. But whether the age-related decline in telomerase expression affects all or selected subsets of T cells *in vivo* and whether it is due to reduced hTERT expression or other post-transcriptional mechanisms are currently unknown.

To understand the role of hTERT/telomerase in T cell differentiation and aging, we measured levels of total hTERT mRNA and ASPs, and telomerase activity in freshly isolated as well as *in vitro* stimulated CD4^+^ and CD8^+^ T_N_, T_CM_, and T_EM_ cells from 111 human subjects (aged from 17 to 85 years old). We report a differentiation dependent decline in hTERT mRNA expression (both full-length and alternative splicing products) and telomerase activity that correlated with their *in vitro* cell proliferative capacity and viability after stimulation. Furthermore, we showed knockdown of hTERT mRNA with an antisense oligo modestly reduced telomerase activity and resulted in decreased proliferation of activated CD4^+^ T_N_ cells. Finally, we found that CD4^+^ T_N_ and T_CM_ cells exhibited an aged-related reduction in activation-induced telomerase activity. Taken together, our results show that T cell differentiation is associated with progressively reduced hTERT expression and telomerase activity, resulting in reductions in cellular proliferative capacity and viability that are further compounded by aging.

## Materials and Methods

### Isolation of Six T Cell Subsets From Human Subjects

Blood was collected from apheresis packs or buffy coats from healthy human adults from the NIA clinic and NIH blood bank under IRB approved protocols. Peripheral blood mononuclear cells (PBMCs) were further isolated by Ficoll gradient centrifugation (GE Health science). CD4^+^ and CD8^+^ T cells were enriched by negative immunomagnetic selection using custom made mouse antibody cocktails and anti-mouse IgG magnetic beads as previously described (32, 33). Enriched CD4^+^ and CD8^+^ T cells were resuspended (5 × 10^6^ cells/ml) in RPMI1640 with 10% Fetal bovine serum and 100 U/mL penicillin-streptomycin and incubated overnight at 37°C and 5% O_2_. The following morning, cells were stained with antibodies against CD4 (OKT4), CD8 (HIT8a), CD45RA (HI100), CD62L (DREG-56) purchased from Biolegend. T_N_ (CD45RA^hi^ CD62L^hi^), T_CM_ (CD45RA^lo^ CD62L^hi^), and T_EM_ (CD45RA^lo^ CD62L^lo^) cells were sorted. The purities of sorted were over 95% and viability were over 99.9% (Figure S1). A portion of sorted cells was immediately frozen or lysed in buffer RLT (Qiagen) as resting cells or stimulated with anti-CD3 and anti-CD28 antibodies (see details below). Due to variations in cell yields and subset proportions among donors, not all subjects had enough cells for all six subsets of resting and activated T cells.

### Stimulation and Culture of T Cell Subsets *in vitro*

Sorted T cell subsets were stimulated with anti-CD3 and anti-CD28 antibodies conjugated to either Dynal beads or microbubbles for 30 min at 37°C with rotation, then placed into 12 well plates at 0.5 × 10^6^ cells/ml and incubated at 37°C. For a standard condition, stimulated cells were harvested at 48 h for hTERT mRNA and telomerase activity measurements. For the 15-day culture experiment, stimulated cells were collected every 3 days for cell count and other analyses including hTERT mRNA, telomerase activity, and cell viability by flow cytometry. Accumulated cell divisions were calculated based on cell counts as the sum of total cell doublings over the course of the culture. Cell viability was determined by dual staining with AnnexinV (Biolegend) and GhostDye (Tonbo Bioscience). Proliferation analysis by CFSE dilution was performed in ModFit (Verity Software House) to calculate proliferation index and cell viability analysis was performed using FlowJo software (FlowJo, LLC). Cell lifespan in culture was determined using cell viability data as follows: death of T cell subsets was defined as the day when the sum of all gates of AnnexinV and GhostDye positive cells reached ≥50%. Kaplan-Meier analysis of T cell subset lifespan was performed in Prism (GraphPad Software).

### Analysis of hTERT Full-Length and ASPs by Quantitative RT-PCR

Total RNA was isolated from cells using the RNeasy Kit (Qiagen). 0.5–5 μg total RNA was used for cDNA synthesis using the Superscript III kit (ThermoFisher) according to the manufacturer's instructions with both oligo dT and random hexamer. Two microliter (one-tenth or less) of the cDNA was used for each qPCR reaction using either the Maxima SYBR Green/ROX qPCR Master Mix (2X) or PowerUp SYBR Green Master Mix (ThermoFisher), according to the manufacturer's suggestions and using an Applied Biosystems 7500 Fast Real-Time PCR System. For detection of total hTERT mRNA, primers hTERT-S and hTERT-AS located within exon 13 and exon 14, respectively, and interrupted by a 3.2 Kb intron, were used under the following cycling conditions: 2 min at 50°C, 10 min (or 2 min) at 95°C, then 40 cycles of 95°C 15 s, 60°C 30 s, with a final dissociation-curve analysis. Data were normalized to ACOX1 which was detected using primers ACOX1-S and ACOX1-AS that span exon 12 and exon 13, interrupted by a 759 bp intron. Detection of genomic DNA was overcome by efficient DNAseI treatment of RNA and/or the short 30 s extension step. For hTERT ASPs, we used previously reported primers (hTERT2164-S and hTERT2620-AS) (34) to amplify four hTERT isoforms simultaneously. The 5′ and 3′ primers are located in exon 5 and exon 9, respectively, of the hTERT mRNA at positions (2167–2187) and (2600–2623) in reference sequence hTERT variant 1 (NM_198253.2). Platinum Taq PCR kit (ThermoFisher) was used with 400 nM primers and 2 μl of cDNA per 20 μl reaction under the following conditions: 94°C for 2 min, then cycled at 94°C for 30 s, 60°C for 30 s, and 72°C for 30 s. In general, 40 and ≤35 cycles of PCR were applied to resting and activated T cells, respectively, to avoid the saturation of amplification. PCR products were run on 2% agarose gels followed by ethidium bromide staining and bands were quantified by densitometry using ImageJ (NIH) or Genesys software (Syngene). A diagram of the qPCR and isoform PCR assays is shown in Figure S2. Sequence information of all primers can be found in Table S4.

### Analysis of Cytokine Expression by Quantitative RT-PCR

cDNA from various experiments were used to measure two growth related cytokines (IL2 and IL21) by qPCR described in the above section, The primer sequences can be found in Table S4.

### Measurement of Telomerase Activity

Telomerase activity was measured as previously described (35). In brief, 0.5 × 10^6^ cells were collected for each sample and lysed at 1 × 10^6^ cells /100 μl in CHAPS lysis solution supplemented with 0.1 mM PMSF. 10,000 cell equivalents of CHAPS lysate were used for telomere synthesis in a 10 μl volume containing the following components: 1X Telomerase reaction buffer (50 mM Tris-OAc at pH 8.5, 1 mM MgCl_2_, 50 mM KOAc, 5 mM β-mercaptoethanol, 10 mM spermidine), 2 mM telomere dNTP mix (dATP, dGTP, dTTP), and 1 μM of PAGE-purified Ts oligo. Two microliter of telomere synthesis solution were used in 15 μl PCR reactions including 1.2 units of Platinum Taq, 1.5 mM MgCl_2_, 0.2 mM dNTP, 200 μM TAMRA-TS, NT, ACX primers, and 1 amole TSNT oligo as an internal quantification control. PCR cycling conditions were as follows: 94°C for 2 min, followed by 32 cycles of 94°C 15 s, 60°C for 20 s. TRAP PCR products were run on non-denaturing 1X TBE buffered 12% PAGE gels and fluorescent TAMRA-TS amplified telomerase products were detected in the Cy3 channel on a Typhoon FLA7000 imaging system (GE Healthcare Life Sciences). Telomerase activity was quantified in ImageJ as the ratio of the first 8 telomerase products to the TSNT internal control. The same lot of Jurkat T cell lysate was used for normalization among different batches of TRAP results.

### Knockdown of hTERT in Human CD4^+^ T_N_ Cells by 2′F-ANA Antisense Oligonucleotide

CD4^+^ T_N_ cells enriched by negative selection were stimulated with anti-CD3/CD28 antibody-conjugated microbubbles for 30 min with rotation. 1 × 10^6^ cells were added in triplicates to 24 well-plates in 2 ml of RPMI containing 2.5 μM hTERT-AS or scrambled oligonucleotide (AUM Biotech) (Table S4) and incubated at 37°C and 5% CO_2_ for 72 h. Cells were harvested at 72 h for viable cell counts by trypan blue exclusion, hTERT mRNA quantification by qPCR, and telomerase activity by TRAP. To assess cell proliferation, cells were labeled with 5 μM CFSE (Biolegend) prior to activation. For viability staining, cells were washed twice in PBS and stained with Annexin V-PE/7-AAD (Biolegend) in Annexin V binding buffer (Biolegend) according to the manufacturer's protocol. Proliferation and viability of cells were analyzed on a CantoII flow cytometer (BD Bioscience). Proliferation indices were generated in ModFit (Verity Software House).

### Statistics

Student's *t* tests, Pearson's correlation, Kaplan-Meier analysis, and 2-way Anova were performed in GraphPad Prism V6.0 and V7.0. For multiple comparisons, Benjamini–Hochberg procedure was computed and presented as FDR with *p* values <0.05 considered as significant.

## Results

### Resting T Cells Express Full-Length hTERT and Its Amounts Decrease With Differentiation

**Figure 1:**
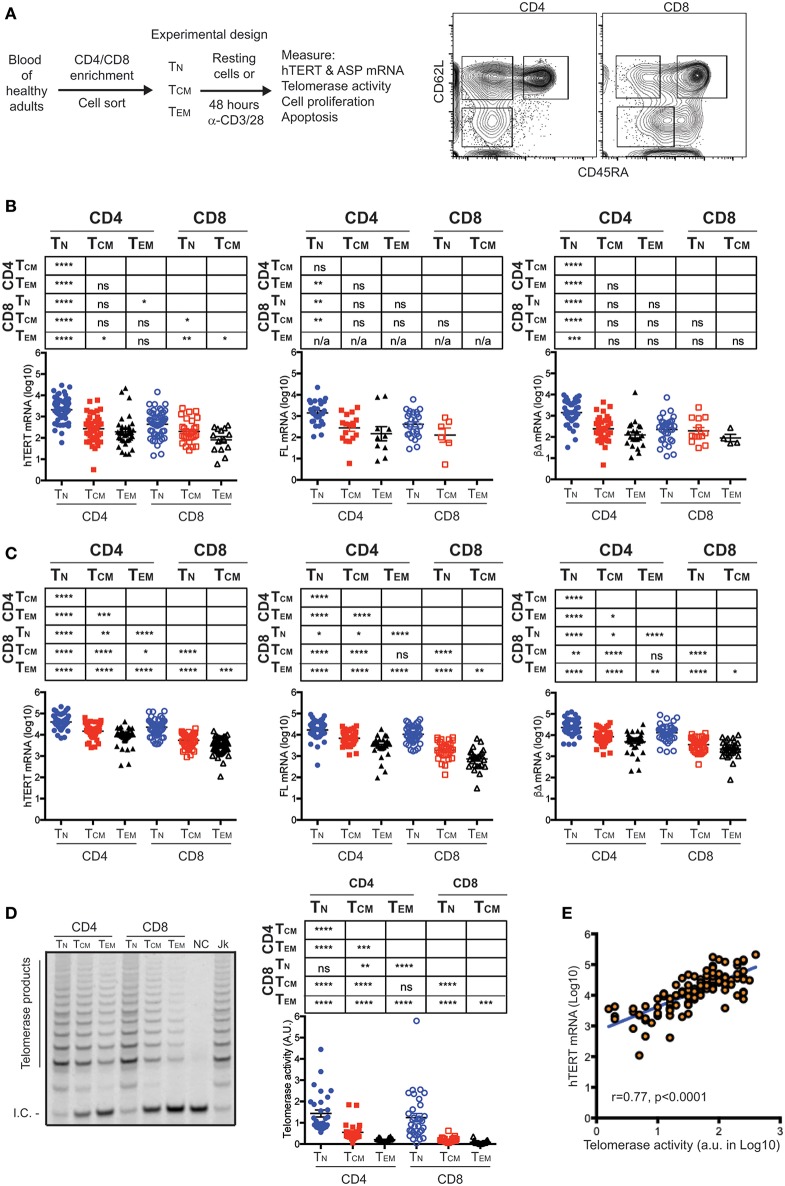
hTERT mRNA expression and telomerase activity decrease with T cell differentiation. **(A)** A schematic of experimental design and a representative FACS plot shows the sorting strategy for isolation of CD4^+^ and CD8^+^ T cell subsets. Subsets of CD4^+^ or CD8^+^ T lymphocytes were sorted from healthy donors as follows: CD62L^+^CD45RA^+^ (naive, T_N_), CD62L^+^CD45RA^−^ (central memory, T_CM_), CD62L^−^CD45RA^−^ (effector memory, T_EM_). **(B)** Total, FL, and βΔ ASP of hTERT mRNA were measured by RT-qPCR in resting T cell subsets and presented from left to right. Unpaired Student's *t*-tests were performed followed by the Benjamini–Hochberg correction. The FDR adjusted *p*-values are presented in the table above the figure and significant differences are marked in red. Each dot represents one individual from a total of 98 individuals and the values of Log_10_ transformed -ΔCT are presented with addition of 5 to make all values positive. **(C)** Total, FL, and βΔ ASP of hTERT mRNA was measured by RT-qPCR in activated T cell subsets. Sorted T cell subsets were stimulated anti-CD3/CD28 antibody for 48 h and total, FL and βΔ ASP of hTERT were quantified by RT-PCR. **(D)** A representative gel image shows telomerase activity measured in one donor by TRAP assay. NC, no template control; Jk, Jurkat positive control. At right, telomerase activity was quantified as the ratio of telomerase products to internal control (I.C.). **(E)** Correlation between the levels of hTERT mRNA and levels of telomerase activity. Stimulated T cell subsets were measured for both hTERT mRNA and telomerase activity (*n* = 118). The relative hTERT and telomerase activity were plotted. Pearson's correlation (*r* = 0.77, *p* < 0.001). Values represent the mean ± SEM (*n* = 45). *≤0.05, **≤0.01, ***≤0.001, and ****≤0.0001 are used for all figures.

To determine whether hTERT mRNA detected in freshly isolated (resting) T cells is functional, we measured hTERT mRNA (total, full-length, and three common ASPs: αΔ, βΔ, and αΔ + βΔ) by quantitative RT-PCR in freshly isolated CD4^+^ and CD8^+^ T_N_, T_CM_, and T_EM_ of 98 healthy human subjects (Figure 1A, Figure S1 and Table S1). We detected both full-length (FL) as well as ASPs (βΔ ASP most common) of hTERT mRNA in freshly isolated CD4^+^ and CD8^+^ T_N_, T_CM_, and T_EM_ cells (Figure 1B and Figure S2). Among a total of 238 resting T cell subset samples (Table S2), the FL hTERT mRNA was detected in 36% of samples. CD4^+^ and CD8^+^ T_N_ had high detectable rates of FL hTERT mRNA, 47 and 46%, respectively, whereas CD8^+^ T_EM_ cells had undetectable FL hTERT mRNA. Unexpectedly, we found the levels of all forms of hTERT displayed a gradual reduction from T_N_ to T_CM_ to T_EM_ in both CD4^+^ and CD8^+^ T cells. The highest level of hTERT mRNA was found in CD4^+^ T_N_ cells and the lowest in CD8^+^ T_EM_ cells. Interestingly, CD4^+^ T cell subsets had significantly higher hTERT mRNA than their corresponding CD8^+^ T cell subsets. Together, these findings suggest that CD4^+^ and CD8^+^ T cells had intrinsically different set points of hTERT expression levels and that T cell differentiation is associated with reduced expression of hTERT mRNA.

### Activation-Upregulated hTERT mRNA and Telomerase Activity Retain the Hierarchy of Their Relative Abundance in Resting T Cell Subsets

To determine whether the differences of hTERT levels in resting T cell subsets are retained post-stimulation, we activated freshly isolated T cell subsets *in vitro* by anti-CD3 and anti-CD28 antibodies for 48 h and measured their hTERT mRNA and telomerase activity. Significant upregulation of hTERT mRNA (total, FL, and βΔ of ASP) was observed in all six T cell subsets after *in vitro* stimulation and the differentiation-related reduction of hTERT mRNA levels were retained as in resting T cells (Figure 1C). Noticeably, the ratio of FL and ASPs of hTERT remains mostly unchanged from resting to activated T cell subsets (Figure 1C and Table S3), suggesting that ASP of hTERT is pegged to the total amount of hTERT mRNA not to T cell differentiation. Next, we wanted to determine whether the different levels of hTERT mRNA are linked to the levels of telomerase enzymatic activity in these T cells. We found that the levels of telomerase activity in activated T cells displayed the same order as hTERT mRNA levels in activated T cell subsets, namely, the highest level of telomerase activity was found in activated CD4^+^ T_N_ cells and the lowest in activated CD8^+^ T_EM_ (Figure 1D) and indeed the levels of hTERT mRNA and telomerase activity was positively correlated in a linear fashion (Figure 1E). These findings suggest that T cell differentiation is associated with the reduction of hTERT mRNA and activation-induced telomerase activity, but activation does not alter the ratio between functional and non-functional hTERT mRNA in all six T cell subsets.

### Higher Levels of hTERT/telomerase Are Linked to Enhanced Cell Proliferation and Viability of T Cells After *in vitro* Stimulation

**Figure 2:**
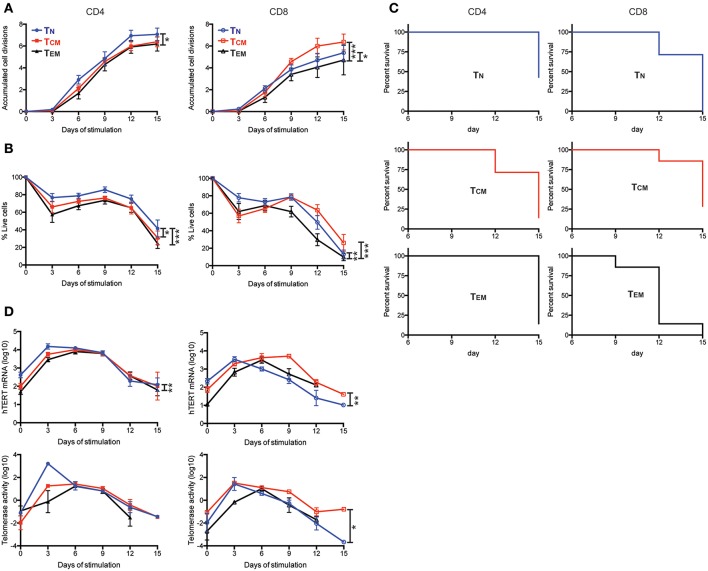
Enhanced cell proliferation and viability are enabled by high levels of hTERT mRNA and telomerase activity. **(A–D)** The indicated T cell subsets were stimulated with anti-CD3/CD28 conjugated microbubbles at day 0 and cultured for the indicated times. **(A)** The average number of accumulated cell divisions (*n* = 9) are shown. Average accumulated cell divisions were calculated from the sum of the log2-transformed fold-increases in number of viable cells harvested over the number of viable cells harvested at the prior time point. **(B)** Averaged percentages of live cells are shown (*n* = 7). Live cells were defined as those negative for AnnexinV and GhostDye as determined by flow cytometry. **(C)** Kaplan-Meier survival analysis of cell subsets from seven subjects during 15-days of culture. CD4^+^ and CD8^+^ T cell subsets are shown in the left and right panels, respectively. Death of the cell culture was defined as when the sum of all gates for AnnexinV^+^ and GhostDye positive cells reached ≥50%. (*n* = 7) **(D)** Total hTERT mRNA measured by RT-qPCR (top panels) and TRAP measurement of telomerase activity (bottom panels) during the 15-days of culture. *p*-values are derived by two-way ANOVA test where *as *p* ≤ 0.05, **as *p* ≤ 0.01, and ***as *p* ≤ 0.001.

To further determine whether the levels of hTERT/telomerase correlated with activation-induced proliferation and cell survival, we stimulated six T cell subsets with anti-CD3 and anti-CD28 antibody *in vitro* and analyzed cell number and survival every 3 days over a 15-day culture after stimulation. We found that CD4^+^ T_N_ cells had the most cell divisions and highest percentage of live cells whereas CD8^+^ T_EM_ cells had the fewest cell divisions and lowest percentages of live cells among six subsets of T cells (Figures 2A,B). In addition, CD4^+^ T_N_ cells from 43% of subjects (3 of 7 donors) had more live cells (viable cells >50%) at the end of the 15-day culture compared to CD8^+^ T_EM_ cells from 0% of subjects had a similar percentage of live cells at the end of 15-day culture (Figure 2C). Interestingly, CD8^+^ T_CM_ cells had less cell expansion than CD8^+^ T_N_ in the first 6 days but surpassed CD8^+^ T_N_ cells in expansion at day 9 of culture. To determine whether hTERT expression and telomerase activity could explain this change, we measured hTERT mRNA and telomerase activity and found that both were higher in CD8^+^ T_N_ than CD8^+^ T_CM_ at day 3, but lower at day 6 and 9 of culture, corresponding closely with their *in vitro* expansion (Figure 2D). We further investigated whether the differences in hTERT/telomerase expression were due in part to the production of T cell growth cytokines IL2 and IL21 and found that there was no statistically significant difference in mRNA levels of IL2 and IL21 among CD4^+^ and CD8^+^ T cell subsets (Figure S3). Collectively, these results show that levels of hTERT mRNA and telomerase activity are closely associated with activation-induced cell proliferation and survival and suggest that T cell differentiation results in reduced proliferative ability.

### Knockdown of hTERT mRNA Levels Result in Reduced CD4^+^ T_N_ Proliferation and Survival *in vitro* in Response to Activation

**Figure 3:**
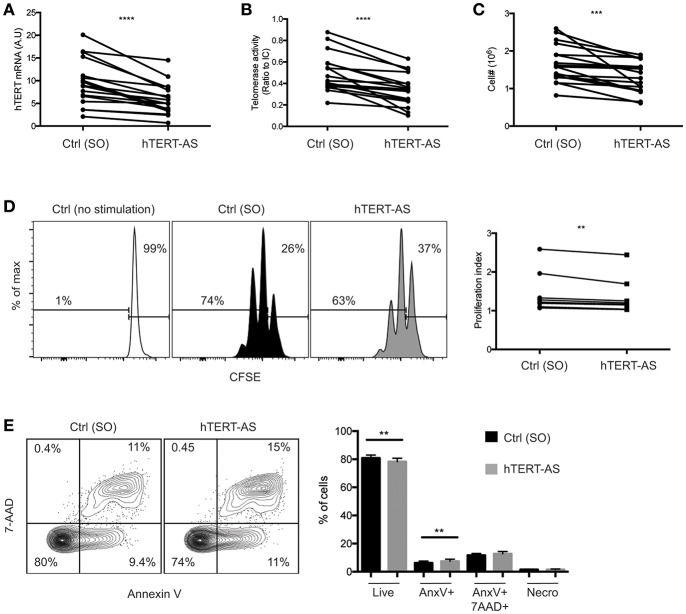
Knockdown of hTERT mRNA leads to increased cell death in activated CD4^+^ T_N_ cells. **(A–E)** CD4^**+**^ T_N_ cells were activated with anti-CD3/CD28 antibody in the presence of scrambled control [Ctrl (SO)] or hTERT-specific antisense oligonucleotide (hTERT-AS). After 72 h, total hTERT mRNA expression was measured by qPCR **(A)**, telomerase activity was measured by TRAP **(B)**, and viable cells were counted by trypan blue exclusion **(C)**. Lines connect the mean value of triplicate wells treated with Ctrl or hTERT-AS. Data are from 17 individual donors from 12 independent experiments. **(D)** A representative graph shows CFSE-labeled CD4^**+**^ T_N_ cells after 72 h of activation in the presence of Ctrl or hTERT-AS antisense oligonucleotide. Freshly isolated CD4^**+**^ T_N_ cells cultured for 72 h in the presence of 20 U/ml IL-2 [Ctrl (no stimulation)] are shown as an undivided control. Change in proliferation index is shown at right. Data are from 8 individual donors from 8 independent experiments. **(E)** Following 72 h of activation in the presence of oligonucleotide, cells were analyzed for live (AnnexinV^−^,7AAD^−^), early apoptosis (AnnexinV^+^,7AAD^−^), late apoptosis (AnnexinV^+^7AAD^+^), and necrosis (AnnexinV^−^,7AAD^+^). Mean percentages of populations are quantified at right. Data are representative of 16 individual donors. Paired Student's *t*-tests were performed between hTERT-AS and control. **p ≤ 0.01, ***p ≤ 0.001, and ****p ≤ 0.0001.

To directly assess the role of hTERT mRNA and telomerase activity in activation-induced T cell proliferation, we knocked down hTERT mRNA using an antisense oligonucleotide for RNase-H mediated mRNA degradation. CD4^+^ T_N_ cells were enriched and activated by anti-CD3 and anti-CD28 antibody in the presence of an hTERT-specific (hTERT-AS) oligonucleotide or a scrambled oligonucleotide (SO) control for 72 h. We confirmed that CD4^+^ T_N_ cells treated with hTERT-AS had significantly reduced levels of hTERT mRNA (average 62%) and telomerase activity (average 58%) compared to the control (Figures 3A,B). More importantly, we observed a modest but significant reduction in cell numbers for hTERT-AS treated CD4^+^ T_N_ cells (average 79%) compared to the control (Figure 3C). To determine whether the reduced cell numbers were due to reduced cell proliferation and/or increased cell apoptosis, we analyzed cell proliferation using CFSE tracking dye and found a significant reduction in cell divisions in hTERT-AS treated CD4^+^ T_N_ cells compared to the control (Figure 3D). Furthermore, we found that hTERT-AS treated CD4^+^ T_N_ cells had a modest reduction in live cells and also a modest increase of early apoptotic cells compared to control treated cells (Figure 3E). We also examined whether knockdown of hTERT expression would affect the growth cytokine IL2 and found a minor decrease of IL2 mRNA under knockdown conditions of some subjects but the difference was not statistically significant (Figure S4). Thus, these results demonstrated that hTERT/telomerase enhances activation-induced proliferation and reduces activation-induced apoptosis of CD4^+^ T_N_ cells.

### Age Reduces hTERT mRNA and Telomerase Activity in Activated CD4^+^ T_N_ and T_CM_ Cells

**Figure 4:**
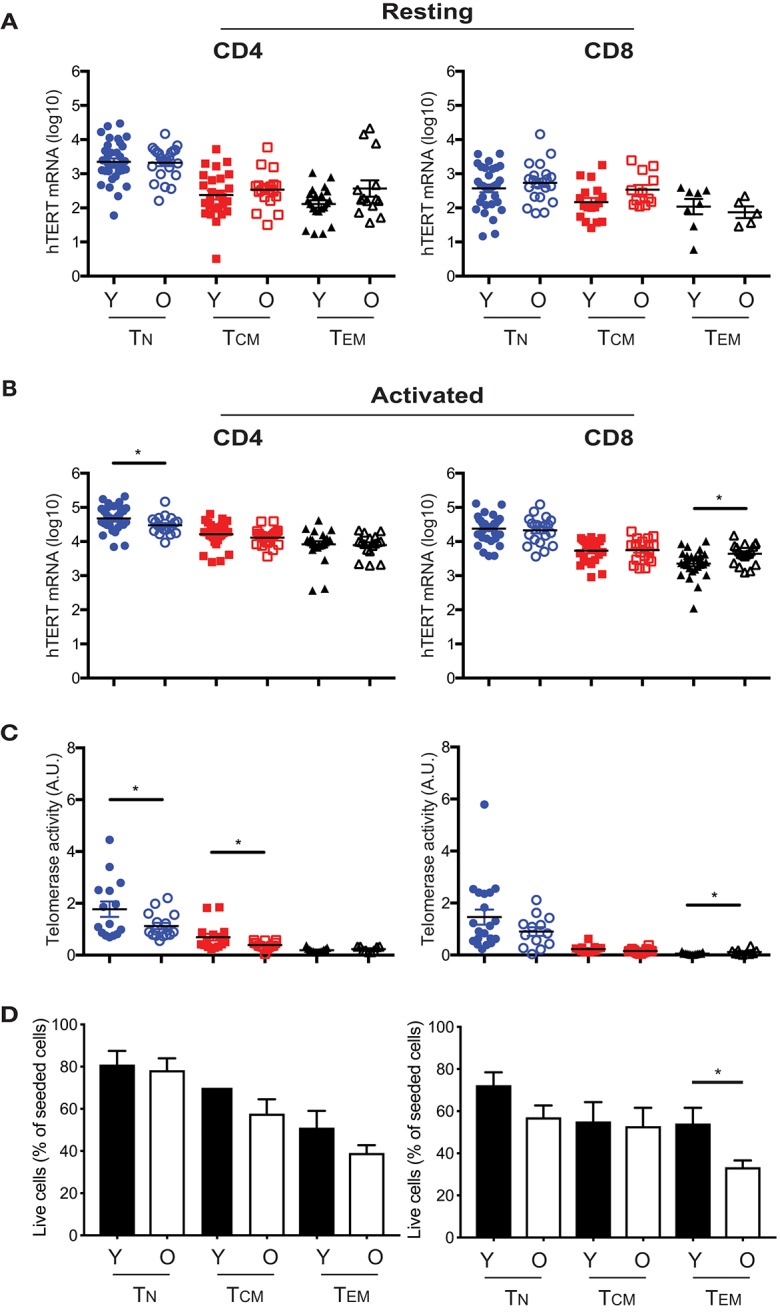
Age-associated alterations in hTERT mRNA and telomerase activity in resting and activated T cell subsets. Comparison of total hTERT mRNA in CD4^+^ and CD8^+^ freshly isolated **(A)** and activated by *in vitro* stimulation with anti-CD3/CD28 antibody for 48 hrs. **(B)** T cell subsets from young (Y, closed symbol) (age ranges 17–40, *n* = 25–37) and old (O, open symbol) (age ranges 68–85, *n* = 16–27) donors. **(C)** Comparison of activation-induced telomerase activity in T cell subsets of young (*n* = 13–20) and old (*n* = 9–16) subjects. Each dot represents one individual. **(D)** Cell recovery after *in vitro* stimulation of six CD4^+^ and CD8^+^ T cell subsets from young and old subjects (Y, *n* = 16; O, *n* = 28). Percentages (mean and SEM) of seeded cells are presented. Values represent the mean ± SEM and *p*-values are derived by unpaired Student's *t*-test. *p ≤ 0.05.

Our previous study showed that activation induced telomerase activity in T cells was reduced in old compared to young human subjects (30). However, it was not known whether this change was a result of reduced hTERT mRNA or other mechanisms post hTERT expression nor whether reduced telomerase activity occurred in all or selected T cell subsets in the old. To address these questions, we compared hTERT mRNA levels and induced telomerase activity between young (≤40 years) and old (≥68 years) human subjects. We found that young subjects did not have higher hTERT mRNA (total, FL, and ASP) in resting T cell subsets than did old subjects (Figure 4A and Figure S5). After activation *in vitro*, the hTERT mRNA in CD4^+^ T_N_ cells was significantly higher in young than in old subjects, but this was reversed in CD8^+^ T_EM_ cells which had significantly higher hTERT mRNA in old than in young subjects (Figure 4B). Comparing activation-induced telomerase activity, we found that activated CD4^+^ T_N_ and CD8^+^ T_EM_ cells had significantly higher telomerase activity in parallel with their hTERT mRNA changes (Figure 4C). As the levels of telomerase activity in CD8^+^ T_EM_ cells were orders of magnitude lower than that of CD4^+^ T_N_ cells, the impact of such a change on telomerase activity of total T cells was negligible. Strikingly, CD4^+^ T_CM_ cells also had significantly higher induced telomerase activity from young than from old subjects without a significant difference in hTERT mRNA levels, suggesting age impairs telomerase expression in T cells of old subjects not only at hTERT mRNA but also subsequent levels. To further determine whether the difference in hTERT/telomerase expression between young and old subjects was correlated with cell growth, we found similar numbers of harvested CD4^+^ T_N_ and T_CM_ cells after stimulation between young and old subjects (Figure 4D). Together, these findings demonstrated that the age-associated reduction of activation-induced telomerase activity affect mainly in CD4^+^ T_N_ and T_CM_ cells which does not appear to be related to their activation-induced growth.

## Discussion

Tight developmental regulation of hTERT/telomerase expression in most normal somatic cells is considered an essential step for preventing uncontrolled growth of differentiated cells (19). Our findings here show that hTERT/telomerase expression is also tightly regulated during differentiation of CD4^+^ and CD8^+^ T cells from naïve to memory cells. As T cell differentiation leads to clonal expansion of selected T cells, limiting telomerase expression in these cells could also be a protective measure for limiting uncontrolled or unwanted T cell clonal expansion that takes away space and resources from other T cells which will be detrimental to the host. This differentiation-coupled regulation is also operating at the level of hTERT transcription with unique features: FL hTERT mRNA is present in resting CD4^+^ and CD8^+^ T cells without measurable telomerase activity and activation up-regulates both hTERT mRNA and telomerase activity in T cells. Interestingly, hTERT ASPs are also detected in both resting and activated T cells but their amounts relative to the FL hTERT remain stable indicating alternative splicing of hTERT is related to the total amount of hTERT mRNA but not T cell differentiation. Furthermore, the level of hTERT/telomerase expression is positively correlated with activation-induced proliferation and cell survival of all six subsets of T cells in response to *in vitro* activation and reducing hTERT levels results in reduced proliferation and increased apoptosis. Collectively, our findings reveal a differentiation-associated progressive decrease in hTERT/telomerase expression in human T cells, which in turn reduces the capacity of activation-induced T cell proliferation.

Previous studies have shown that resting human T cells from blood exhibit low to undetectable levels of telomerase activity despite the presence of hTERT mRNA (14–16). Jalink et al. analyzed a limited number of human subjects (*n* = 6) and showed that only the βΔ ASP, not FL hTERT mRNA, was present in resting T cells (18). Here we found CD4^+^ and CD8^+^ T_N_ cells from approximately half of study subjects had FL hTERT mRNA but no detectable FL hTERT mRNA in CD8^+^ T_EM_ cells from any study subjects. Although undetectable telomerase activity in the presence of FL hTERT in resting T cells might be explained by the lack of activated kinases required for telomerase activity that are downstream of TCR, such as activated AKT (36), or the presence of PP2A phosphatase that inhibits telomerase activity via dephosphorylating both AKT and hTERT (37), the presence of both FL and ASPs in 30% of five T cell subsets (excluding CD8^+^ T_EM_) raises another possibility that the lack of telomerase activity in these resting T cells could also be due to the ASPs outcompeting the FL hTERT for hTERC binding resulting in non-functional telomerase complexes (38, 39). However, it is worth noting that the measurements of hTERT mRNA and telomerase activity were carried out in bulk cells. It is currently unknown whether hTERT FL and ASPs are present in a single resting T cell, or whether individual cells exclusively express either FL or ASP. To resolve this, assessing hTERT at the single cell level will be needed, but the sparse expression of hTERT mRNA in freshly isolated T cells is currently a major hurdle that will require assays of improved sensitivity.

T cell generation and differentiation from lineage committed precursor cells are precisely regulated processes (40). During naïve to effector to memory T cell differentiation, several key transcription factors have been reported in the control of differentiation (41–46). Our findings show that hTERT exhibits reduced expression during T cell differentiation in both CD4^+^ and CD8^+^ T cells and reduction of telomerase activity is associated with decreased activation-induced proliferation and increased apoptosis *in vitro*. The functions of telomerase in T cell differentiation could potentially involve both telomere maintenance and extratelomeric roles such as cell survival (47–49). The influence of telomerase on T cell survival is further observed here in differentiated T cell subsets in *in vitro* culture as well as in knockdown of hTERT/telomerase in CD4^+^ T_N_ cells. Roth et al. have shown that blocked endogenous hTERT expression by ectopic expression of dominant-negative hTERT resulted in decreased lifespan of T cell clones *in vitro* (24). More recently, Zhdanov et al. used a bacterially derived telomerase inhibitor (*Rhodospirillum rubrum* L-asparaginase) and show that reduced telomerase results in telomere shortening, reduced lifespan, and increased apoptosis in CD4^+^ T cells *in vitro* (50). Higher telomerase activity in mature CD4^+^ compared to mature CD8^+^ single positive thymocytes has been reported (14). Here we find that peripheral CD4^+^ T cell subsets expressed more hTERT mRNA and higher activation-induced telomerase activity than their CD8^+^ T cell counterparts. This suggests that there is a lineage difference in hTERT/telomerase expression between CD4^+^ and CD8^+^ T cells from thymus to periphery. Intriguingly, CD4^+^ T cells have higher levels of activation-induced telomerase activity and longer replicative lifespan than do CD8^+^ T cells *in vitro*. Whether and how such differences impact the functions of CD4^+^ and CD8^+^ T cells *in vivo* remains to be determined. Importantly, how hTERT expression is regulated during T cell differentiation will require further study.

T cells with shorter telomeres are found in old humans, which has been attributed to cumulative telomere loss due to cell divisions over time (28). Our recent report of reduced activation-induced telomerase activity in T cells of old compared to young humans (30) suggests that reduced telomerase enzymatic activity also contributes to telomere shortening with age. Here we show age-associated alterations in activation-induced telomerase activity in T cell subsets. CD4^+^ T_N_ and T_CM_ cells have significantly reduced telomerase activity in response to *in vitro* activation in old compared to young humans whereas CD8^+^ T_EM_ cells have significantly elevated telomerase activity in old compared to young humans. While induced telomerase activities in CD4^+^ T_N_ and CD8^+^ T_EM_ cells are closely correlated with hTERT mRNA, CD4^+^ T_CM_ also showed reduced telomerase activity in old compared to young subjects without significant reduction of hTERT mRNA. This suggests that aging affects activation-induced telomerase not only at hTERT transcription but also in the subsequent levels of telomerase formation. Such age-related alteration of telomerase in activated T cells may explain the impaired activation-induced proliferation of T cells found in old subjects (51, 52). The difference of hTERT/telomerase expression was not due to better proliferation of young than old subjects. Thus, the precise changes that occur with age underlying the alteration of telomerase induction in CD4^+^ T_N_, T_CM_, and CD8^+^ T_EM_ cells in old humans remain unclear. A better understanding of hTERT/telomerase regulation and its change in old humans could provide a basis for strategies to enhance telomerase activity for improving T cell function in old humans and in T cell-based therapeutics.

## Data Availability

All datasets generated for this study are included in the manuscript and/or the Supplementary Files.

## Ethics Statement

This study was carried out in accordance with the guidelines of the National Institute on Aging and approved by the Institutional Review Board. Study subjects were participants of the Baltimore Longitudinal Study of Aging and NIH Blood Bank and gave written informed consent.

## Author Contributions

MP and JK conducted experiments and analyzed data. N-LC, JA, FD, QY, and IZ developed reagents and performed experiments. MP and NW designed the studies and wrote the manuscript.

### Conflict of Interest Statement

The authors declare that the research was conducted in the absence of any commercial or financial relationships that could be construed as a potential conflict of interest. The handling editor declared a shared affiliation, though no other collaboration, with the authors.
