# Supplementary material for: Human T Cell Differentiation Negatively Regulates Telomerase Expression Resulting in Reduced Activation-Induced Proliferation and Survival
Source: Front Immunol. 2019 Aug 21;10:1993. doi: 10.3389/fimmu.2019.01993 (PMC6712505; doi:10.3389/fimmu.2019.01993)
Supplement: Supplementary file 1 [file Data_Sheet_1.docx]

Supplementary Material


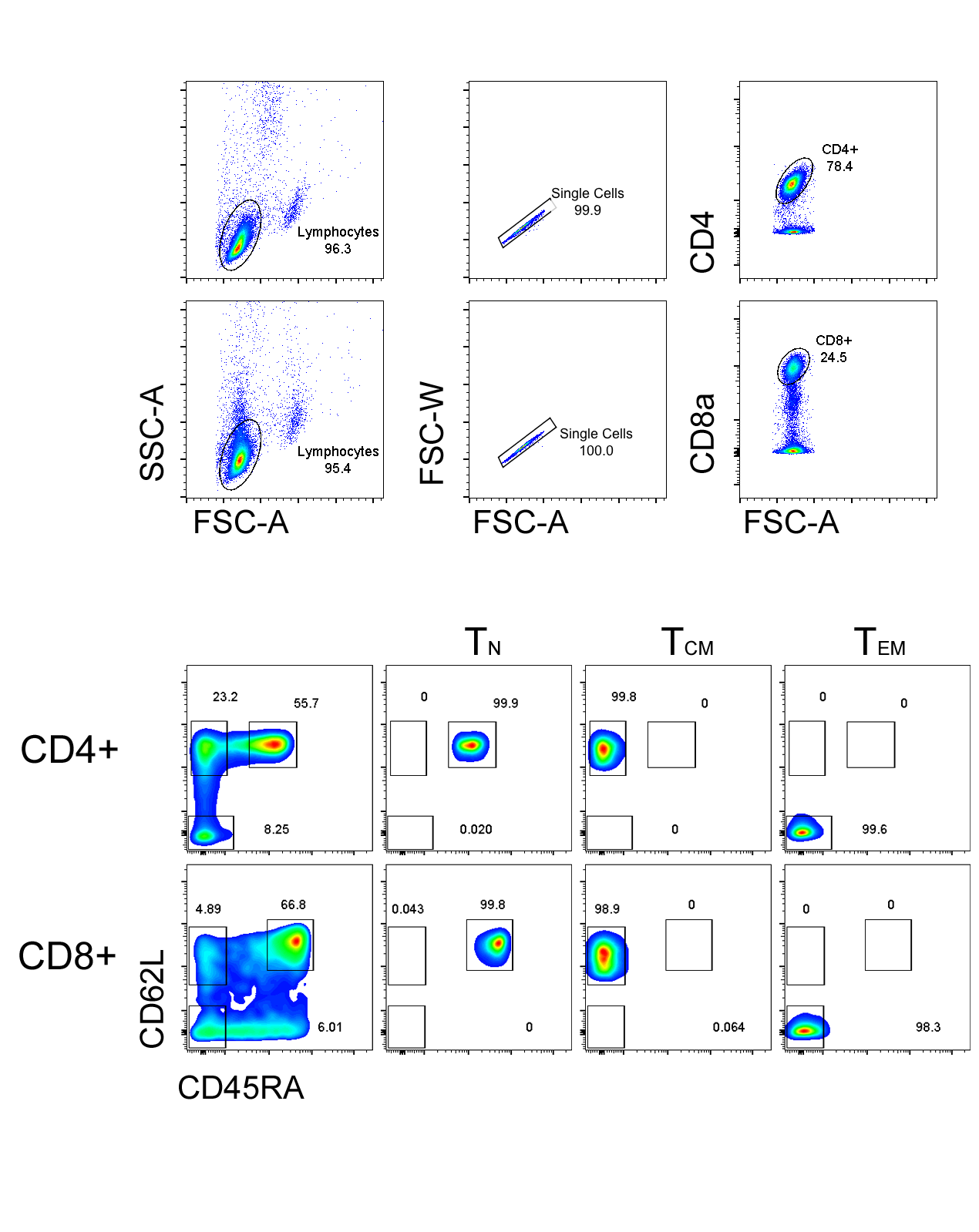


Figure S1. Gating strategy of isolating T_N_, T_CM_ and T_EM_ from CD4^+^ and CD8^+^ T cells. Gating strategy of enriched T cells to CD4^+^ and CD8^+^ T cells (top panel) and gating strategy of T_N_, T_CM_ and T_EM_ of CD4^+^ and CD8^+^ T cells (bottom panel) along with the purity analysis of sorted cells.


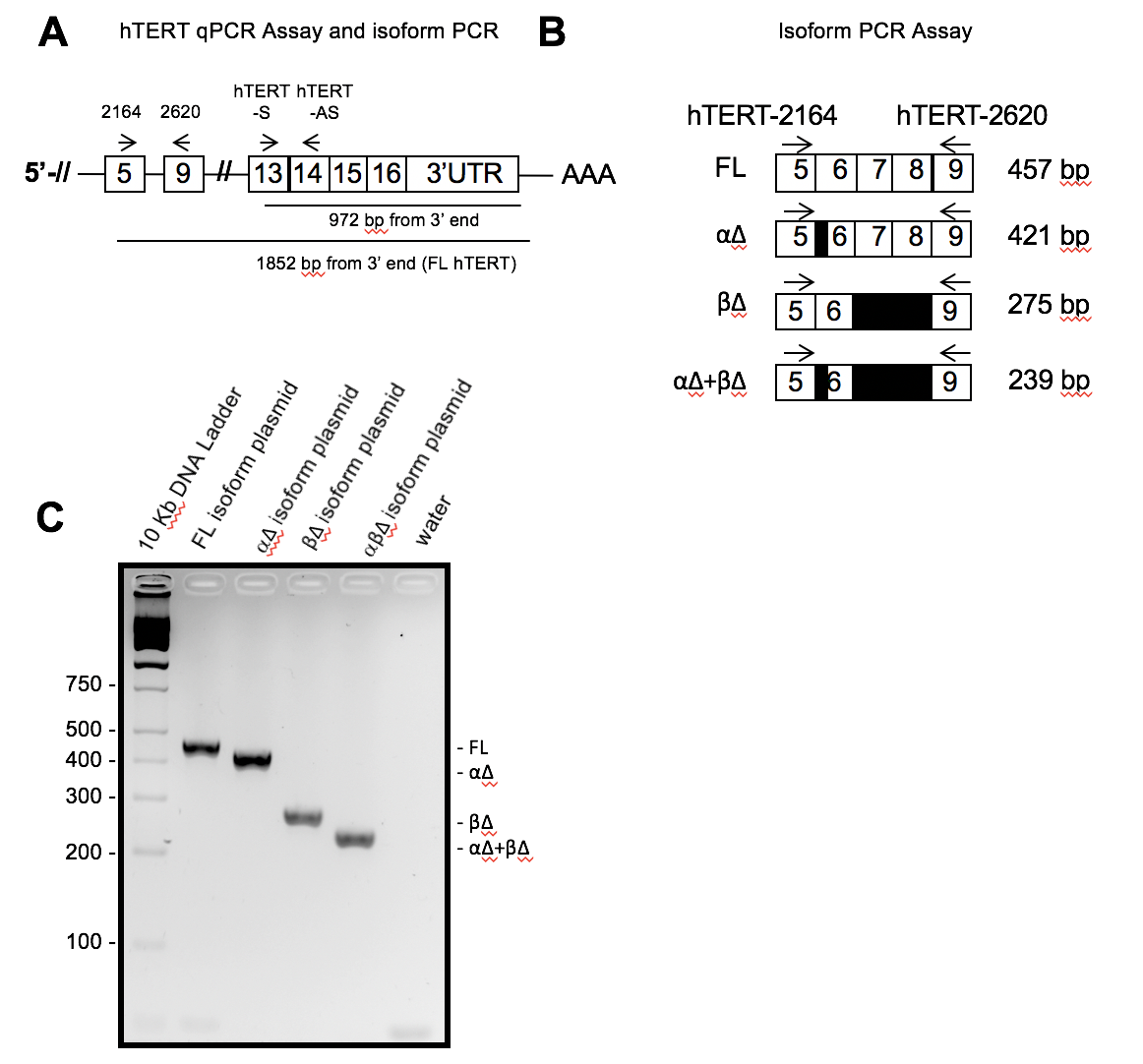


Figure S2. Description of hTERT qPCR and ASP-specific PCR assays. (A) qPCR and ASP-specific PCR primers are indicated on a simplified hTERT mRNA. Exons are indicated by boxes. (B) ASP-specific primers are shown in exons 5 and 9 which generate the 4 ASPs indicated. Filled boxes indicate regions excised by alternative splicing. Amplicon sizes in base-pairs (bp) are shown at right. (C) Plasmids harboring the cloned ASPs were amplified using primers hTERT-2164/2620 and analyzed on ethidium bromide stained 2% agarose gels.

**Figure S3. IL2 and IL21 mRNA levels in T_N_, T_CM_ and T_EM_ of CD4^+^ and CD8^+^ T cells during 15-day culture after anti-CD3/CD28 stimulation in vitro**. (A) IL2 mRNA (B) IL21 mRNA normalized to ACOX1 in CD4^+^ and CD8^+^ T cell subsets. Two-way Anova test showed no statistical significance among the three subsets.

Figure S4. IL2 mRNA levels in CD4^+^ T_N_ cells in the presence of hTERT knockdown or control oligo. IL2 mRNA was measured by RT-PCR and normalized to ACOX1. No significant difference by paired Student’s t test.


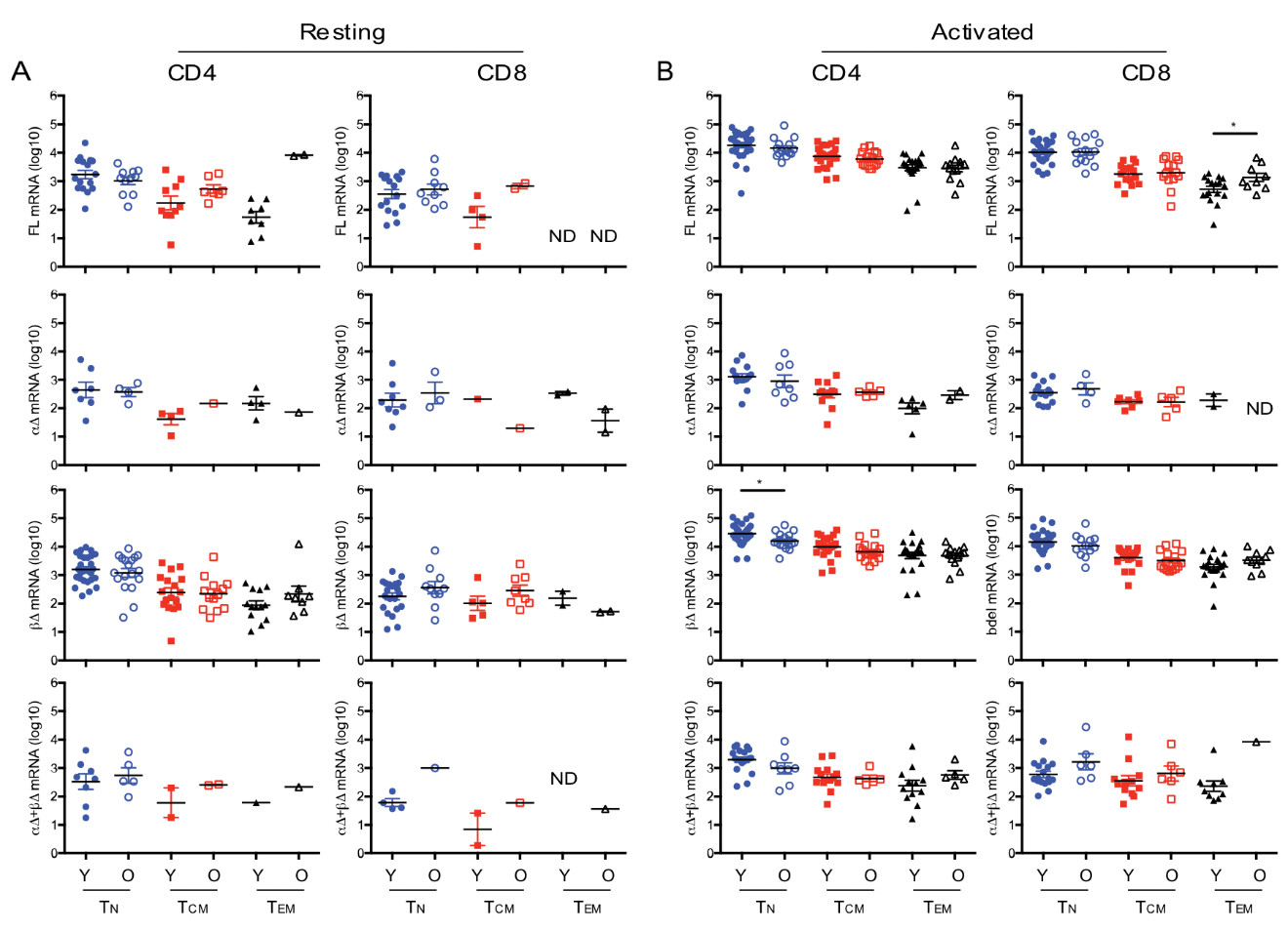


Figure S5. Comparison of hTERT FL and ASPs in young and old donors. (A) Resting and (B) activated CD4^+^ and CD8^+^ T cell subsets were measured by RT-qPCR for expression of FL, αΔ, βΔ, and αΔ+βΔ. Young (Y, closed symbol) (age ranges 17-40) and old (O, open symbol) (age ranges 68-85) donors are shown.

Table S1. Summary of genders and ages of donors used in study, ordered by increasing age.


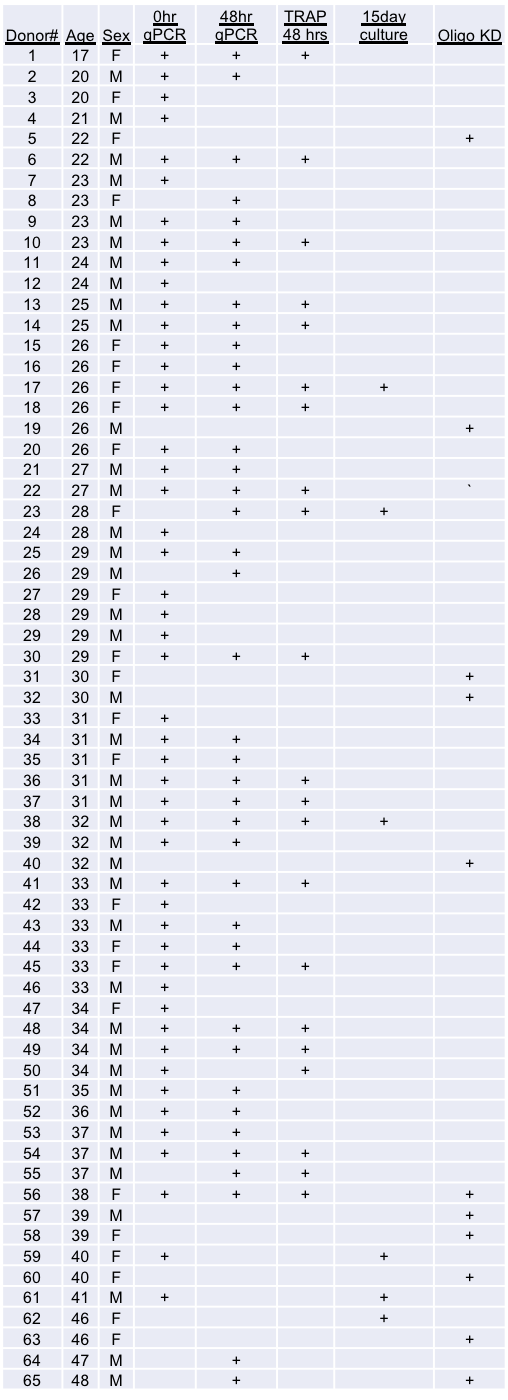

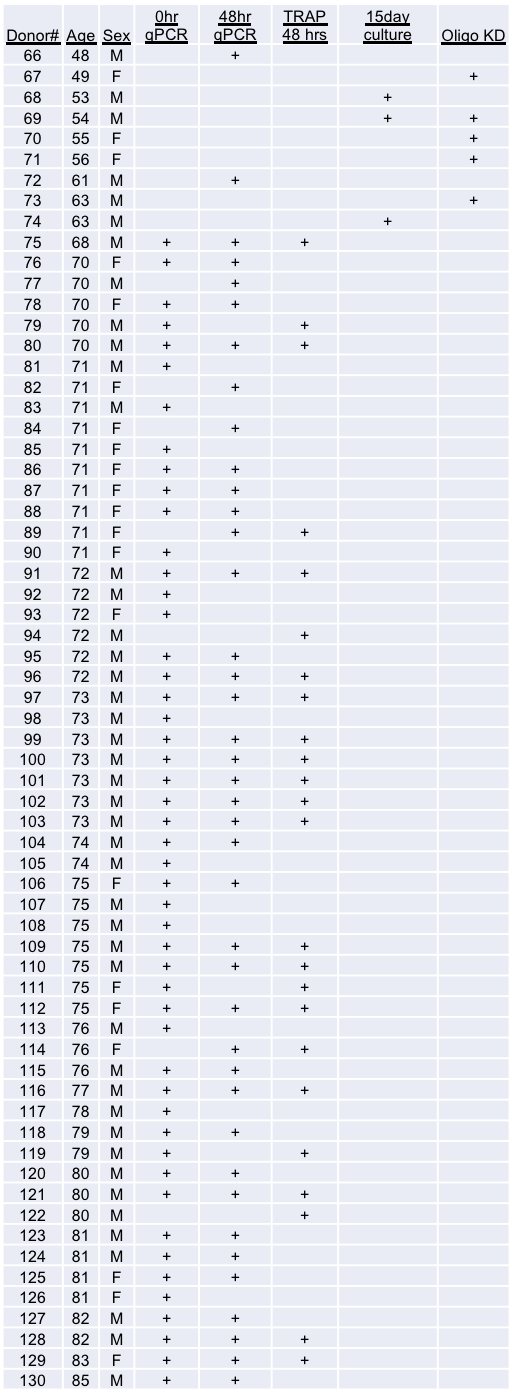


Table S2. Summary of alternatively spliced product detection in resting T cell subsets.


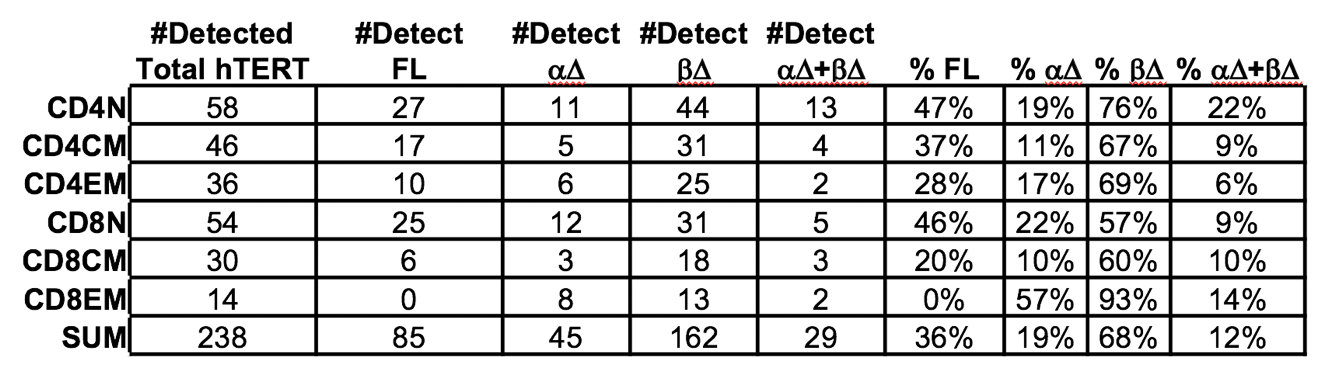


Table S3. FL/βΔ ratios in resting and activated T cell subsets.

**
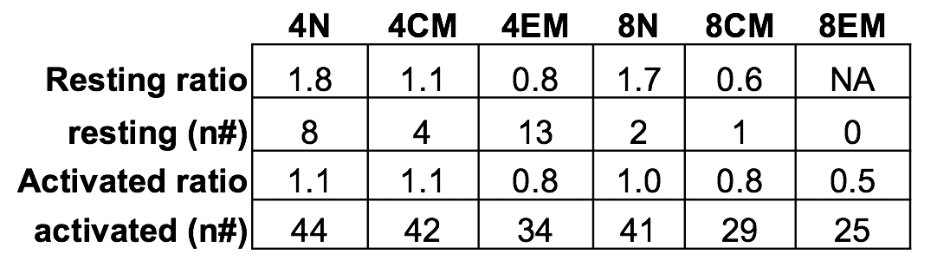
**

Table S4. Sequences of oligonucleotides used in this study.

| **Primer name** | **Sequence** | **Assay** | **Target** |
| --- | --- | --- | --- |
| TS | AATCCGTCGAGCAGAGTT | TRAP |  |
| NT | ATCGCTTCTCGGCCTTTT | TRAP |  |
| ACX | GCGCGGCTTACCCTTACCCTTACCCTAACC | TRAP |  |
| TSNT | AATCCGTCGAGCAGAGTTAAAAGGCCGAGAAGCGAT | TRAP |  |
| hTERT2164-S | GCCTGAGCTGTACTTTGTCAA | Isoform PCR | hTERT |
| hTERT2620-AS | CGCAAACAGCTTGTTCTCCATGTC | Isoform PCR | hTERT |
| hTERT-S | GTGTGCACCAACATCTACAAG | qRT-PCR | hTERT |
| hTERT-AS | CATCAGCAAGTTTGGAAGAACC | qRT-PCR | hTERT |
| ACOX1-S | CCATTCAAGCTGTCTTAAGGAG | qRT-PCR | ACOX1 |
| ACOX1-AS | TTGTGTAATCTGAGGCTCTGTC | qRT-PCR | ACOX1 |
| IL2-S | AAGAATCCCAAACTCACCAGGAT | qRT-PCR | IL2 |
| IL2-AS | TAGACACTGAAGCTGTTTCAGTTCTG | qRT-PCR | IL2 |
| IL21-S | GCCAGCTCCAGAAGATGTAGAGA | qRT-PCR | IL21 |
| IL21-AS | GGGCCTTCTGAAAACAGGAAA | qRT-PCR | IL21 |
| 2’F-ANA hTERT-AS | CAUCAGCCAGTGCAGGAAC | FANA Knockdown | hTERT |
